# Evaluation of clinical applicability of automated liver parenchyma segmentation of multi-center magnetic resonance images

**DOI:** 10.1016/j.ejro.2022.100448

**Published:** 2022-11-02

**Authors:** Varatharajan Nainamalai, Pravda Jith Ray Prasad, Egidijus Pelanis, Bjørn Edwin, Fritz Albregtsen, Ole Jakob Elle, Rahul P. Kumar

**Affiliations:** a The Intervention Centre, Oslo University Hospital - Rikshospitalet, Sognsvannsveien 20, 0372 Oslo, Norway; b Department of Informatics, University of Oslo, Oslo, Norway; c Institute of Clinical Medicine, University of Oslo, Oslo, Norway; d Department of Hepato-Pancreatic-Biliary Surgery, Oslo University Hospital - Rikshospitalet, Oslo, Norway; e Institute for Cancer Genetics and Informatics, Oslo University Hospital, Oslo, Norway

**Keywords:** Magnetic resonance images, Liver parenchyma, Deep learning, Segmentation, Level set, Clinical applicability

## Abstract

**Purpose:**

Automated algorithms for liver parenchyma segmentation can be used to create patient-specific models (PSM) that assist clinicians in surgery planning. In this work, we analyze the clinical applicability of automated deep learning methods together with level set post-processing for liver segmentation in contrast-enhanced T1-weighted magnetic resonance images.

**Methods:**

UNet variants with/without attention gate, multiple loss functions, and level set post-processing were used in the workflow. A multi-center, multi-vendor dataset from Oslo laparoscopic versus open liver resection for colorectal liver metastasis clinical trial is used in our study. The dataset of 150 volumes is divided as 81:25:25:19 corresponding to train:validation:test:clinical evaluation respectively. We evaluate the clinical use, time to edit automated segmentation, tumor regions, boundary leakage, and over-and-under segmentations of predictions.

**Results:**

The deep learning algorithm shows a mean Dice score of 0.9696 in liver segmentation, and we also examined the potential of post-processing to improve the PSMs. The time to create clinical use segmentations of level set post-processed predictions shows a median time of 16 min which is 2 min less than deep learning inferences. The intra-observer variations between manually corrected deep learning and level set post-processed segmentations show a 3% variation in the Dice score. The clinical evaluation shows that 7 out of 19 cases of both deep learning and level set post-processed segmentations contain all required anatomy and pathology, and hence these results could be used without any manual corrections.

**Conclusions:**

The level set post-processing reduces the time to create clinical standard segmentations, and over-and-under segmentations to a certain extent. The time advantage greatly supports clinicians to spend their valuable time with patients.

## 1 Introduction

Colorectal cancer is considered the third-most common cancer in the world and the second leading in mortality [1], [2], [3]. About 50% of the patients with colorectal cancer develops colorectal liver metastases [4], [5], and resection of metastases is considered to be the curative option [6]. Though resection can be done both by open surgery and laparoscopic surgery, laparoscopic liver surgery has more advantages in postoperative benefits with reduced blood loss, shorter hospital stays, etc. [7], [8], [9]. Furthermore, laparoscopic parenchyma-sparing liver resection allows radical resection with maximum preservation of the liver that contributes to decreased risk of postoperative liver failure [10]. This requires preparation with extensive planning related to the assessment of resection margin and segmentation of liver anatomy from medical images by the creation of patient-specific 3D models (PSM) [11], [12]. Computed tomography (CT) is considered the choice for imaging, but magnetic resonance (MR) gained its popularity for improved lesion-to-liver contrast and non-ionizing radiation, etc. [13], [14], [15].

Artificial intelligence (AI) - based deep learning methods may reduce the workload of creating these PSMs by performing the tasks automatically and allowing the clinicians to interpret the segmentation with less time. Nowadays, with the availability of medical data and high computational power, there is increased usability of AI techniques in segmentation and surgery planning. Effective clinical utilization of AI-created PSMs still needs to be corrected to a certain extent and requires validation by clinicians. It is important to analyze the clinical relevancy of liver segmentation using automated algorithms, especially the time spend to create clinically acceptable segmentation, etc. [16].

The convolutional neural network models are playing an important role in medical images [17]. Xio Han [18], [19] has used a novel deep convolutional neural network, and achieved a high score in Liver Tumor Segmentation (LiTS) challenge 2017. Christ et al. [20] used a cascaded UNet model on diffusion weighted-MR images, and achieved 87% Dice score. Fu et al. [21] proposed a novel method using a trio of CNNs, and achieved a Dice score of 95.3% in the liver segmentation task. Valindria et al. [22] investigated using a dual-stream encoder-decoder architecture for abdominal multiple organ segmentation on unpaired CT and MR images, and achieved a Dice score of 94.3% in the liver. The effectiveness of UNet is proven in comparison with multi-atlas segmentation in T1-weighted 3D-SPGR images, Owler et al. [23] concluded that UNet approaches were more effective in obtaining a median Dice of 97% using 3D UNet architecture. Chen et al.[24] proposed a 2D UNet with densely connected blocks for multi-organ segmentation on multi-slice MR images, and obtained the Dice score of 96.3% in liver metrics. Couteaux et al. [25] used single and multi-channel networks which involves a comparison of segmentation between paired-unregistered T1 and T2 weighted MR images using 3D-UNet architecture, and achieved Dice score 96.1% for T1 and 93.8% for T2 sequences respectively. Given the consideration of the potential impact of UNet in literature, we decided to use a variant of UNet with shape-based prior knowledge.

Despite the fact that the CNN model delivers results with higher accuracy, they are dependent on the training features learned by the model from the available dataset. On the other hand, shape-based techniques utilizing prior knowledge have promising results in the clinical domain. The level set method, as a part of the active contour model family, has evolved over the past 20 years with a higher impact on medical image segmentation [26]. To address the problem of over-segmentation in MR images, Chen et al. [27] proposed a method by employing a multiple initialization level set method achieving a true positive fraction of 95%. Middleton et al. [28] described combining neural networks together with snake models to perform segmentation in MR images, and conclude that spatial inputs can contribute to marginal improvements in segmentation performance and poor segmentation can be improved by modifying active contour models. Using a region-based level set function, Omar Ibrahim Alirr [29] implemented a fully convolutional neural network for liver segmentation in CT images, and achieved a Dice score of 95.6% in the LiTS dataset. Shu et al. [30] proposed a method for liver CT images based on a level set with energy functional including the data fitting term, the length term and the bound term, and achieved a Dice score of 97.57%. All these successful level set methods confirm their effectiveness in medical images and a potential domain of research.

There are many techniques based on automated or semi-automated methods for segmentations available nowadays. Further, how effectively these segmentations are utilized for visualization and surgical planning, the time required to modify clinically relevant 3D PSMs, prospectively clinician’s experience, etc., require more studies to implement 3D PSMs in everyday clinical routine [31], [32], [33]. In this work, we study the clinical relevancy of liver segmentation on multi-center MR images (T1-sequence) using a deep learning algorithm, and with level set post-processing. We also measure the time required to create clinically acceptable segmentation for deep learning, and deep learning initialized level set post-processed segmentations. The novelty of this article includes multi-center MR data, extensive parameter optimization of the level set method, and comprehensive clinical analysis.

This paper is organized as follows: in Section 2, a detailed description of the dataset, ground truth delineation criteria, proposed liver segmentation model with data pre- and post-processing particulars together with evaluation metrics are provided. In subsequent sections, numerical and subjective results of both deep learning and level set post-processing followed by a clinical discussion of these results are presented in Section 3. In Section 4, final observations of the results obtained, conclusions, and limitations of the proposed study are presented.

## 2 Materials and methods

### 2.1 Data acquisition

Our study includes contrast-enhanced T1-weighted MR images (150 volumes) gathered through Oslo laparoscopic versus open liver resection for colorectal liver metastases clinical trial study [8], [9], [34], [35]. The study was performed in accordance with the ethical standards of the institutional and/or national research committee and with the 1964 Helsinki declaration and its later amendments or comparable ethical standards.

The database consists of medical images obtained from various hospitals in Norway, therefore was performed with both Siemens and Philips MRI machines on 7 different models. The T1-weighted MR images have been acquired using various protocols and combinations of Dixon, eThrive, Vibe with various different timings between contrast injection and imaging. Hence, the database includes images with a high level of variability. The image set has 150 anonymized MR image volumes and is converted into NIfTI format.

#### 2.1.1 Ground truth segmentation

The ground truth manual segmentation creation for the dataset was performed by a medical doctor (over 4-year medical image post-processing experience) with 3D Slicer [36]. In Slicer, several basic and advanced segmentation tools and techniques were used to make the ground truth. These are paint, erase, margin, grow from seed, smoothing, scissors, islands, and logical operators with a threshold to mention some. Delineation criteria are the following: Ground truth includes liver tissue as a whole organ including lesions, all liver vessels, and bile ducts adjoined to the liver parenchyma viewed on the axial plane. Segmentation does not include the gallbladder and the vena-cava is segmented on one additional axial slice cranially.

### 2.2 Image pre-processing and augmentations

The dataset of 131 volumes is taken out of 150 volumes, and divided into 81, 25, and 25 volumes for train, validation, and test dataset respectively. The remaining 19 volumes of the dataset were used for testing the clinical applicability of the deep learning segmented results. The volume dimensions are varying as 176–640, 168–560, 60–150, and voxel spacing varies as 0.56–1.95, 0.56–1.95, 1.6–3 mm in axial, coronal, and sagittal planes respectively of the dataset.

An input volume of the dataset contains the minimum voxel intensity zero and the maximum voxel value depending on each volume which is related to the reconstruction algorithm MR imaging scanner. The huge variations lead to difficulties in learning features during training and make converge slower. The min-max normalization scales the intensity between 0 and 1 which makes the better network learning, and gradient descent algorithm converge faster. We have used intensity scaling, contrast adjustment, Gaussian smoothness, Gaussian sharpening, flipping, rotation, and elastic 3D deformation as augmentations on the fly during the training of the network with a probability of 0.3. The parameter values in each augmentation choice are randomly chosen at each epoch. The flipping axis (random flip with respect to axis 0 or 1 or 2) and rotation (− 0.4 radian to +0.4 radians) are considered.

### 2.3 Network

In this study, a simple UNet variant model is used. We have considered UNet with 16 channels in the first layer (denoted by UNet_16_), UNet with 32 channels in the first layer (denoted UNet_32_), and an attention gate with each UNets model.

**Fig. 1:**
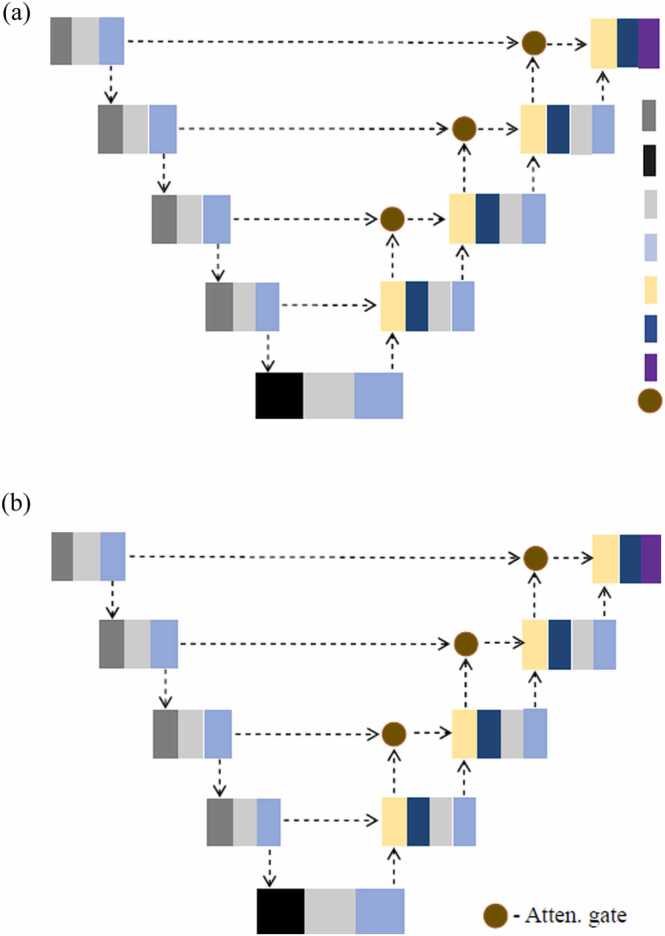
Deep learning models from UNet convolutional neural network variants (a) UNet, and (b) UNet with attention gate in the decoder (Conv. str-2 - Convolution with stride 2, Tr. conv. str-2 - Transpose convolution with stride 2).

Fig. 1(a) explains the UNet architecture of convolution layers with stride 2 which helps to down-sample and up-sample spatial resolutions. The network consists of five levels with encoder-decoder layers. In the encoder, we represent the convolutional layers with 3 × 3 × 3 kernel with stride 2 as light gray layers. The pale white and light blue layers represent batch normalization and PReLU activation functions, respectively. The black layer represents the convolutional layers of 3 × 3 × 3 kernel with stride 1. The thick blue color blocks represent transpose convolutions of 3 × 3 × 3 kernel with stride 2, and the yellow color blocks represent concatenations. The dashed lines with arrows connect different layers of the network. We applied Argmax to extract one-channel network prediction.

We used the attention gate in the UNet_16_ and UNet_32_ decoder part of the network, shown as brown circles in Fig. 1(b). It consists of convolution with 1 × 1 × 1 kernel for both the skip connections and up-convolution channels followed by additive attention with relu activation function. Then a convolution with 1 × 1 × 1 kernel and sigmoid activation function is used. Finally, it has a 3D grid-attention re-sampler that makes a 3D volume.

For training the deep learning model, an Intel(R) Core (TM) i7–7700 K CPU 4.20 GHz (8 cores), 64 GB RAM, and NVIDIA Geforce GTX 1080 Ti - PCIE - 11 GB of Video RAM was used. The networks were trained using Novagrad optimizer with a learning rate of 0.001. The NovoGrad Optimizer is a stochastic normalized gradient descent algorithm that includes the information of layer-wise second moments, computes the first moment with gradients normalized with layer-wise second moments, and decouples weight decay. In comparison with the Adam optimizer, NovoGrad takes less memory and is more numerically stable [37].

### 2.4 Loss functions

The Dice loss, generalized Dice loss, and Tversky loss functions are used to analyze the network performance. The Dice similarity coefficient (DSC) is used to measure spatial overlap between manual segmentation and predicted image segmentation. If $p_{i}$ and ${\overset{ˆ}{p}}_{i}$ are voxels of expected and predicted label class values respectively, the Dice loss (DL) function (based on the Dice similarity coefficient) can be written as

(1) $$\mathrm{DL}=1-\frac{2\sum_{i=1}^{N}p_{i}{\overset{ˆ}{p}}_{i}+\epsilon }{\sum_{i=1}^{N}p_{i}^{2}+\sum_{i=1}^{N}{\overset{ˆ}{p}}_{i}^{2}+\epsilon },$$

where *ϵ* = 10^−6^ and *N* takes all voxels of the manual segmentation [38], [39]. The generalized Dice loss (GDL) obtained from generalized Dice score (used to evaluate multi-class *K* label segmentation with a single score) can be utilized for training deep CNN models described by

(2) $$\mathrm{GDL}=1-2\frac{\sum_{l=1}^{K}w_{l}\sum_{i=1}^{N}p_{li}{\overset{ˆ}{p}}_{li}}{\sum_{l=1}^{K}w_{l}\sum_{i=1}^{N}\left(p_{li}+{\overset{ˆ}{p}}_{li}\right)},$$

where $w_{l}=\frac{1}{\sum_{n=1}^{N}{\overset{ˆ}{p}}_{ln}}$ is the weight [38], [39], [40].

The Tversky similarity Index (TI) is balancing false positive (FP) and false negative (FN), given more weighted FN than FP, defined as

(3) $$\mathrm{TI}=\frac{\mathrm{TP}}{\mathrm{TP}+\alpha \mathrm{FP}+\beta \mathrm{FN}},$$

where *α* + *β* = 1 and *β* > *α*. Hence, these conditions leads to *α*, *β* ≰ 0.5. If *α*, *β* = 0.5, the Tversky similarity index becomes the Dice similarity score. The Tversky loss (TL) can be derived as TL = 1 − TI, where TI is the Tversky index [41]. In the case of *α* and *β* with the restriction *β* > *α* and *α* + *β* = 1, gives the parameter choices for (*α*, *β*) = {(0.4, 0.6), (0.3, 0.7), (0.2, 0.8), (0.1, 0.9)} (change in *α* and *β* are 0.1), represented Tversky losses as TL46, TL37, TL28, TL19.

### 2.5 Data post-processing

A level set algorithm as post-processing to refine the deep learning liver parenchyma segmentation was used. We consider the distance regularized level set evolution (DRLSE) which is a variant of the variational level set formulation [42]. The DRLSE algorithm possesses a potential function that acts as a distance regularization term, and it helps the gradient magnitude of the level set function to a minimum point. In our case, we consider a double-well potential function that has two minima. Also, DRLSE algorithm has an external energy term, that helps to move the zero-level contour to the desired location. The gradient flow, diffusion equation, can be obtained by exterimizing the total energy integral [42] as

(4) $$\frac{\partial \phi }{\partial t}=\mu \mathrm{div}\left(d_{p}\left(|\nabla \phi |\right)\nabla \phi \right)+\lambda \delta_{\epsilon }\left(\phi \right)\mathrm{div}\left(g\frac{\nabla \phi }{|\nabla \phi |}\right)+\alpha g\delta_{\epsilon }\left(\phi \right),$$

where the initial level set function given by *ϕ*(*x*, 0), *μd*_*p*_(∣ ∇ *ϕ*∣) is the diffusion coefficient, *δ* is the Dirac delta function, *λ* > 0, α∈R, and *g* is an edge indicator function defined by $\frac{1}{1+|\nabla G_{\sigma }*I{|}^{2}}$, *G*_*σ*_ is a Gaussian kernel with a standard deviation *σ*, and *I* is an image on a domain Ω. The *α* and *λ* are the coefficients of area and length terms. Using DRLSE algorithm we iteratively proceed to the steady solution by following the steepest descent direction.

### 2.6 Evaluation metrics

In general, the evaluation of segmentation results can be classified using a confusion matrix with true positive (TP), false positive (FP), false negative (FN), and true negative (TN). We used the following performance metric for the evaluation: The Dice similarity coefficient (DSC or Dice score) is a measure of the spatial overlap between the predicted segmentation and the manual segmentation written as

(5) $$\mathrm{DSC}=\frac{2\mathrm{TP}}{2\mathrm{TP + FP + FN}}.$$

The Jaccard index (JAC) is defined as the intersection over the union of the predicted segmentation and the manual segmentation defined by

(6) $$\mathrm{JAC}=\frac{\mathrm{TP}}{\mathrm{TP}+\mathrm{FP}+\mathrm{FN}}.$$

Over segmentation (OS) measures the overlapping of the prediction and the complement of label voxels in the ground truth over a union of ground truth and prediction defined by

(7) $$\mathrm{OS}=\frac{2|\overset{¯}{\mathrm{GT}}\cap \mathrm{Pred}|}{|\mathrm{GT}|+|\mathrm{Pred}|},$$

where GT is the ground truth, Pred represents the prediction, and $\overset{¯}{\mathrm{GT}}$ complement of a class in the manual segmentation [43], [44]. Under segmentation (US) measures overlapping of the complement of the prediction ($\overset{¯}{\mathrm{Pred}}$) and the ground truth over the union of ground truth and prediction defined by

(8) $$\mathrm{US}=\frac{2|\mathrm{GT}\cap \overset{¯}{\mathrm{Pred}}|}{|\mathrm{GT}|+|\mathrm{Pred}|}.$$

The Hausdorff Distance (HD) is a spatial-distance-based measure that is used to evaluate dissimilarity between two segmentation surfaces. We consider modified Hausdorff distance (95 percentile) to remove a small subset of outlier voxels. For two finite point sets *A* and *B*, the 95 percentile Hausdorff Distance can be defined as

(9) $${\mathrm{HD}}_{95}\left(A,B\right)=\max\left(h\left(A,B\right),h\left(A,B\right)\right),$$

where $h\left(A,B\right)=\underset{a\in A}{\max}\underset{b\in B}{\min}||a-b||$ is the directed Hausdorff distance, the norm ∣∣. ∣∣ represents the Euclidean distance between the ground truth *A* and prediction *B* surfaces respectively [45], [46].

### 2.7 Clinical evaluation

A representative set of 19 volumes of MR images were chosen and excluded from any other parts of this work to be used purely for clinical evaluation. The deep learning network model corresponds to the best Dice score of the test dataset to infer the 19 volumes of the clinical evaluation dataset were considered. The clinical evaluation set of MR images has similar characteristics and is balanced (various manufacturers, machine models, slice thickness, lesion localization, and sizes) as the one used for the deep learning network training, validation, and test data sets. We divide the clinical evaluation into two parts clinical usability and review, and segmentation correction.

#### 2.7.1 Clinical usability and review

A couple of meetings, consisting of at least one radiologist and one surgeon, were gathered to review the deep learning results and level-set processed results. The 19 volumes of clinical evaluation MR images, deep learning results, and level set inferences were shown side by side to the blinded review team. The following were analyzed and carried out during the meeting: Firstly, the focus of the discussion was on the accuracy of 3D model, evaluating the liver surface, OS and US, and overall understanding of the liver. Secondly, a detailed examination conducted on the axial view of the MR image was performed along with (deep learning with and without level set) segmentation overlaid with high transparency. This procedure is to evaluate and focus on the segmentation of liver parenchyma, vessels, and lesions, and to identify locations of OS and US. Finally, for each case, a group decision had to be made if segmentations were complete and after future sub-classification of internal anatomical structures could be used for liver surgery planning without any manual corrections. We collected their comments throughout the meeting and finally summarized them into key observations.

#### 2.7.2 Segmentation correction

Manual corrections were performed on the results of deep learning inferences and level set function results of 19 volumes of the clinical evaluation dataset. The segmentation corrections were performed using 3D Slicer with the help of various tools available, including addition, removal, island removal, filling holes, median smoothing, etc. The time required to make necessary changes to the segmentation was recorded from the first change until the review and acceptance of the result. Initially, the deep learning inferences were corrected manually to meet the defined standard of segmentation. Then, the level set function results were edited manually after a blindfold period of five weeks break. The metrics for deep learning (with and without level set) inferences were evaluated against manually corrected segmentation of (deep learning with and without level set) each set. In addition to this, we have estimated intra-observer variations between manually corrected deep learning and level set segmentation to measure clinically acceptable variations for this rator.

## 3 Results

### 3.1 Deep learning results

In this study, we considered a UNet variant and UNet with attention-gate network models. We set 300 epochs for training with early stopping as 25 epochs for all experiments, and hence the training will be stopped when there is no improvement in the learning. The parameters corresponding to the best Dice score of the validation dataset were stored, and it was used to infer the test dataset. Table 1 shows the evaluation of 25 volumes test dataset metrics of the UNet_16_ and UNet_32_ and attention gate models with different losses.

**Table 1:** Average metric values of test dataset of 25 volumes using (i) UNet_16_ (first block) (ii) UNet_32_ (second block) (iii) UNet_16_ with attention gate (Third block) (iv) UNet_32_ with attention gate (fourth block), with different loss functions. Results from UNet_32_ with TL19 loss is accessed for level set experiments.

Network	Loss	DSC	JAC	OS	US	HD_95_
UNet_16_	DL	0.9612 ± 0.0136	0.9255 ± 0.0248	0.0218 ± 0.0238	0.0559 ± 0.0242	3.6158 ± 3.5482
	GDL	0.9579 ± 0.0155	0.9197 ± 0.0277	0.0214 ± 0.0307	0.0628 ± 0.0229	3.7245 ± 3.6924
	TL46	0.9656 ± 0.0104	0.9336 ± 0.0193	**0.0161** ± 0.0162	0.0528 ± 0.0194	3.1974 ± 2.9255
	TL37	0.968 ± 0.0107	0.9382 ± 0.0198	0.0216 ± 0.0186	0.0424 ± 0.0197	3.1383 ± 2.921
	TL28	0.9668 ± 0.015	0.936 ± 0.0269	0.0273 ± 0.0296	0.0392 ± 0.019	3.5011 ± 3.4614
	TL19	**0.9688** ± 0.011	**0.9397** ± 0.0203	0.037 ± 0.023	**0.0254** ± 0.0143	**2.9778** ± 1.8407
**UNet**_32_	DL	0.9623 ± 0.015	0.9277 ± 0.0269	0.022 ± 0.028	0.0534 ± 0.0193	2.9746 ± 2.7522
	GDL	0.9627 ± 0.0125	0.9283 ± 0.0229	**0.0187** ± 0.0172	0.0559 ± 0.0248	2.854 ± 1.9512
	TL46	0.9683 ± 0.0124	0.9389 ± 0.0227	0.0241 ± 0.0224	0.0392 ± 0.0164	3.0974 ± 2.9941
	TL37	0.9678 ± 0.0116	0.9378 ± 0.0214	0.0206 ± 0.0189	0.0439 ± 0.0181	2.857 ± 2.3179
	TL28	0.9692 ± 0.0125	0.9404 ± 0.023	0.0208 ± 0.0193	0.0409 ± 0.0174	**2.6133** ± 2.4184
	**TL19**	**0.9696** ± 0.0118	**0.9413** ± 0.0217	0.0311 ± 0.0236	**0.0296** ± 0.0133	3.2787 ± 2.7023
Att. gateUNet_16_	DL	0.9358 ± 0.0259	0.8804 ± 0.044	0.029 ± 0.0506	0.0994 ± 0.0409	6.7862 ± 7.0749
	GDL	**0.9507** ± 0.0184	0.9065 ± 0.0327	**0.0202** ± 0.0276	0.0784 ± 0.0359	5.6354 ± 4.9625
	TL46	**0.9507** ± 0.0183	**0.9066** ± 0.0324	0.0273 ± 0.0361	0.0712 ± 0.0301	**5.4712** ± 4.9985
	TL37	0.9479 ± 0.0244	0.9018 ± 0.0420	0.0359 ± 0.0489	0.0684 ± 0.0366	6.2078 ± 6.9105
	TL28	0.9343 ± 0.0421	0.8793 ± 0.0675	0.0883 ± 0.0916	**0.0430** ± 0.0216	12.1457 ± 12.932
	TL19	0.8911 ± 0.0491	0.8068 ± 0.0765	0.1697 ± 0.1065	0.0482 ± 0.0200	19.4041 ± 9.8893
Att. gateUNet_32_	DL	0.9438 ± 0.015	0.894 ± 0.0266	0.0175 ± 0.0212	0.0949 ± 0.0321	5.3777 ± 4.2915
	GDL	0.9437 ± 0.0189	0.8939 ± 0.0334	**0.0134** ± 0.0157	0.0992 ± 0.0386	**5.2408** ± 3.9102
	TL46	0.9352 ± 0.0214	0.879 ± 0.0374	0.0365 ± 0.0418	0.0932 ± 0.038	8.0229 ± 6.3321
	TL37	0.9400 ± 0.018	0.8872 ± 0.0315	0.0364 ± 0.0378	0.0837 ± 0.0313	7.3583 ± 6.1879
	TL28	**0.9501** ± 0.0282	**0.9062** ± 0.0470	0.0574 ± 0.0594	0.0423 ± 0.0236	6.6902 ± 7.1561
	TL19	0.9254 ± 0.0400	0.8635 ± 0.0652	0.1106 ± 0.0875	**0.0386** ± 0.0208	12.1541 ± 10.7049

For the UNet_16_, the TL19 show the higher Dice score, less under-segmentation, and less Hausdorff distance. The FP weight *α* = 0.4 and the FN weight *β* = 0.6 show the less OS than other *α*, *β*. For the UNet_32_, the TL19 show the highest Dice score of 0.9696, Jaccard, and less under segmentation. The Hausdorff distance is less for the TL28 model. Less OS has been observed with the generalized Dice loss model. The best scores in Table 1 are highlighted in bold letters.

We also investigated the performance of UNet_16_ and UNet_32_ with the attention gate in the encoder part of the neural network. We observed that UNet_16_ and attention gate with generalized Dice loss function and TL46 shows a higher Dice score of 0.9507. The UNet_32_ with attention gate and TL28 model shows higher Dice, and Jaccard scores. We observed that UNet_32_ with the TL19 loss, without attention gate, shows a higher Dice score. Hence, the inferences of the test dataset of 25 volumes from the TL19 network model are used further for the level set optimization.

### 3.2 Level set results

The test dataset of 25 vol MR images was considered as images for the level set optimization. The test dataset segmentation corresponding to the best Dice score of the deep learning model UNet_32_ with loss function TL19 was used as initial level set functions. Our main focus in this work lies in the optimization of parameters of the partial differential equation (4). The choice of values of these parameters [42] used as the base values for our optimization. In this work, we consider the time step Δ*t* = 1, the Dirac delta function parameter *ϵ* = 0.2, and the Gaussian kernel parameter *σ* = 0.2. To observe the effect of parameter selection in terms of Dice, accuracy, OS, and US, we experimented with several parameter choices. The inner and outer iteration spatial evolution considered, as an ordered pair, (*x*, *x*) such that *x* ∈ {5, 15, …, 65}. The value of *α* = [ − 3, − 7] and *λ* = [3,7] with varying steps Δ*α* = − 1 and Δ*λ* = 1. Our initial level set function which is obtained from deep learning segmentation lies within the liver domain, hence we considered negative *α* values.

**Fig. 2:**
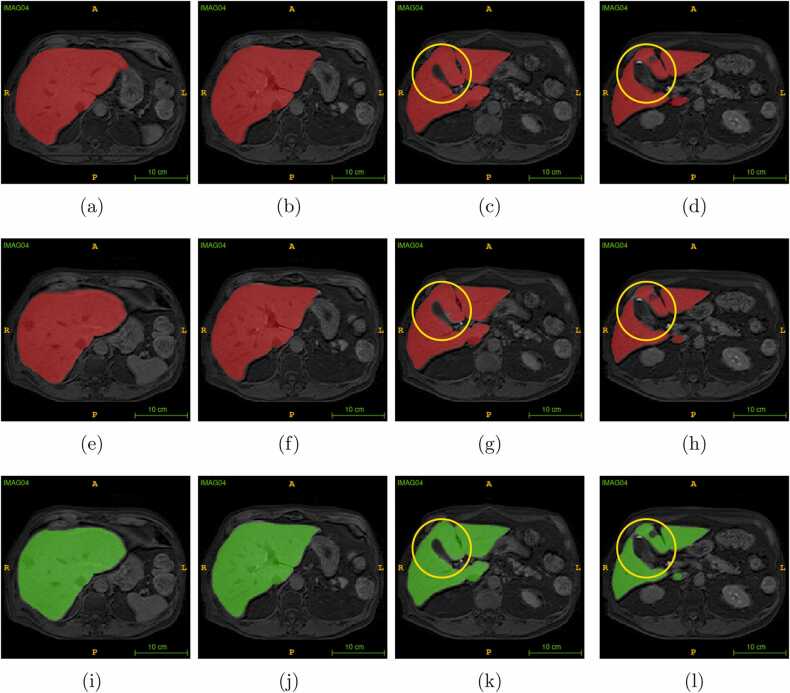
2D axial view of the (a)-(d) ground truth (e)-(h) deep learning results, and (i)-(l) corresponding level set results, of the highest Dice score image volume of the test dataset. The yellow circles show the region where changes are observed from the ground truth.

**Fig. 3:**
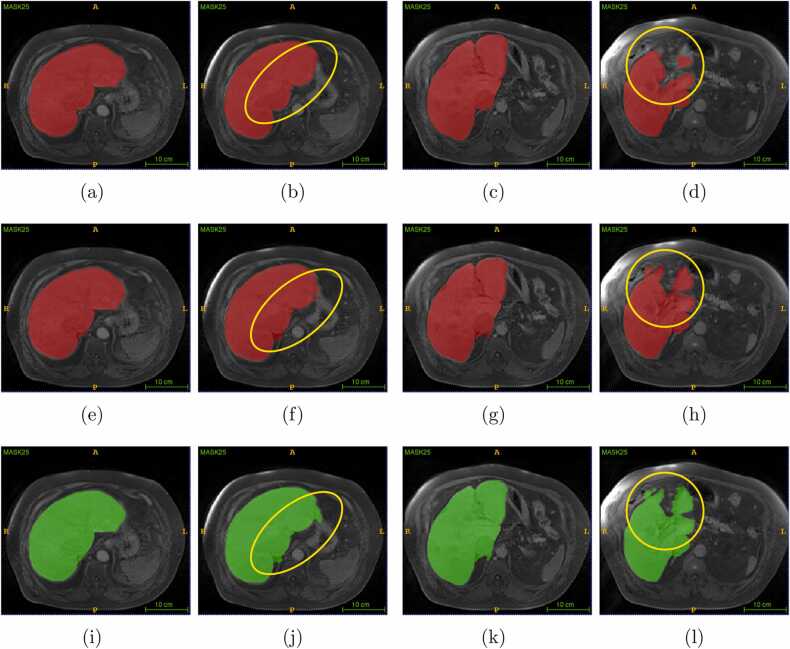
2D axial view of the (a)-(d) ground truth (e)-(h) deep learning results, and (i)-(l) corresponding level set results, of the lowest Dice score image volume of the test dataset. The yellow circles show the region where changes are observed from the ground truth.

**Fig. 4:**
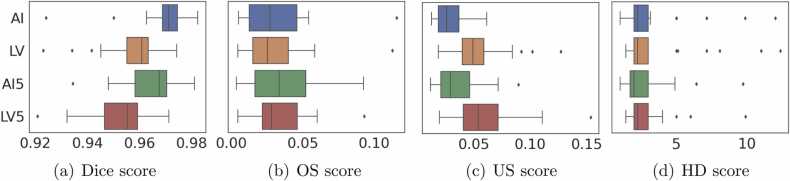
(a)-(d) Comparison of metrics distribution of deep learning, level set prediction, and 5 × 5 × 5 voxels subtracted all over the surface of deep learning and level set predictions, denoted as AI5, LV5 respectively, of the test dataset.

First, we have conducted level set post-processing experiments on slices in axial, sagittal, and coronal planes independently with the same parameters. We noticed that the axial plane shows a higher Dice score than other planes. Then, we used axial plane images for the level set optimization. We observed that *α* = − 5, *λ* = 5, inner iterations = 45 and outer iterations = 25 shows optimum results. In the level set experiments, we obtained the best Dice score of 0.9743 and the lowest Dice score of 0.9231, shown in Table 2. In deep learning-based segmentation, we obtained the best Dice score of 0.9824 and the lowest Dice score of 0.9241. The best and lowest Dice score volumes from the test dataset were compared with the ground truth and level set algorithm results are shown in Figs. 2 and 3. The level set post-processing shows less OS than deep learning results, and other metrics are inferior to the deep learning scores, shown in Fig. 4. We also removed 5 voxel cubes around the edge surface of liver parenchyma of deep learning and level set post-processed segmentations, denoted AI5 and LV5. We expect that border removal can reveal more information about OS and US. Fig. 4 shows the validation results of AI5 and LV5 evaluated against AI, and LV results. The dice and US scores had a greater impact, but less with OS and HD scores.

**Table 2:** Comparison of test dataset of 25 vol metrics of deep learning prediction and level set processed deep learning segmentation scores.

	Deep learning segmentation scores					Level set post-processed segmentation scores				
VolID	Dice	JAC	OS	US	HD_95_	Dice	JAC	OS	US	HD_95_
1	0.9812	0.9632	0.0126	0.0249	1.41	0.9616	0.926	0.0091	0.0678	1.73
2	0.9712	0.944	0.0094	0.0483	5	0.9743	0.9498	0.0151	0.0364	5.1
3	0.9732	0.9478	0.0103	0.0433	9.85	0.9693	0.9404	0.0095	0.052	12.37
4	0.9824	0.9654	0.0057	0.0295	1	0.9614	0.9257	0.0054	0.0718	1.41
5	0.975	0.9512	0.0263	0.0237	2	0.9656	0.9335	0.0224	0.0464	2
6	0.9797	0.9602	0.0234	0.0172	2	0.9731	0.9476	0.0243	0.0295	2.45
7	0.9737	0.9488	0.034	0.0186	2	0.9704	0.9426	0.0406	0.0185	2
8	0.981	0.9627	0.0066	0.0314	1	0.9552	0.9143	0.0053	0.0843	1.73
9	0.97	0.9418	0.0271	0.0328	5	0.9585	0.9203	0.0416	0.0414	8.06
10	0.9501	0.905	0.0541	0.0456	7.14	0.9452	0.8961	0.0584	0.0512	7.14
11	0.9745	0.9503	0.0141	0.0369	2	0.964	0.9306	0.0265	0.0454	5
12	0.9668	0.9357	0.0051	0.0614	2.24	0.9341	0.8764	0.0045	0.1273	2.24
13	0.9727	0.9468	0.0269	0.0278	3	0.9615	0.9259	0.023	0.054	2.45
14	0.9699	0.9415	0.0364	0.0239	2.24	0.9573	0.918	0.0312	0.0543	2.24
15	0.9715	0.9446	0.0311	0.0259	3	0.9628	0.9283	0.0253	0.0491	3
16	0.9627	0.9281	0.0359	0.0387	3	0.955	0.9138	0.0312	0.0589	3
17	0.9635	0.9295	0.0184	0.0547	2.45	0.9416	0.8896	0.015	0.1019	3
18	0.969	0.9398	0.0461	0.016	2	0.9602	0.9234	0.0389	0.0407	2
19	0.9692	0.9403	0.0492	0.0124	3	0.9609	0.9248	0.04	0.0382	2.24
20	0.9694	0.9406	0.0485	0.0127	3.16	0.9601	0.9233	0.0401	0.0397	3
21	0.9735	0.9484	0.0284	0.0246	1.41	0.9634	0.9294	0.0234	0.0498	1.73
22	0.9688	0.9395	0.0462	0.0162	2.24	0.9626	0.928	0.0425	0.0322	2
23	0.9659	0.934	0.0527	0.0156	2.83	0.9599	0.923	0.0487	0.0314	2.24
24	0.9241	0.859	0.1159	0.0359	12	0.9231	0.8572	0.1129	0.041	11
25	0.9822	0.965	0.0127	0.0229	1	0.951	0.9066	0.0058	0.0921	1.73
mean	0.9696	0.9413	0.0311	0.0296	3.2787	0.9581	0.9198	0.0296	0.0542	3.6343
STD	0.0118	0.0217	0.0236	0.0133	2.7023	0.0117	0.0213	0.023	0.0249	2.9495

### 3.3 Study of clinical use of results

#### 3.3.1 Clinical evaluation

**Fig. 5:**
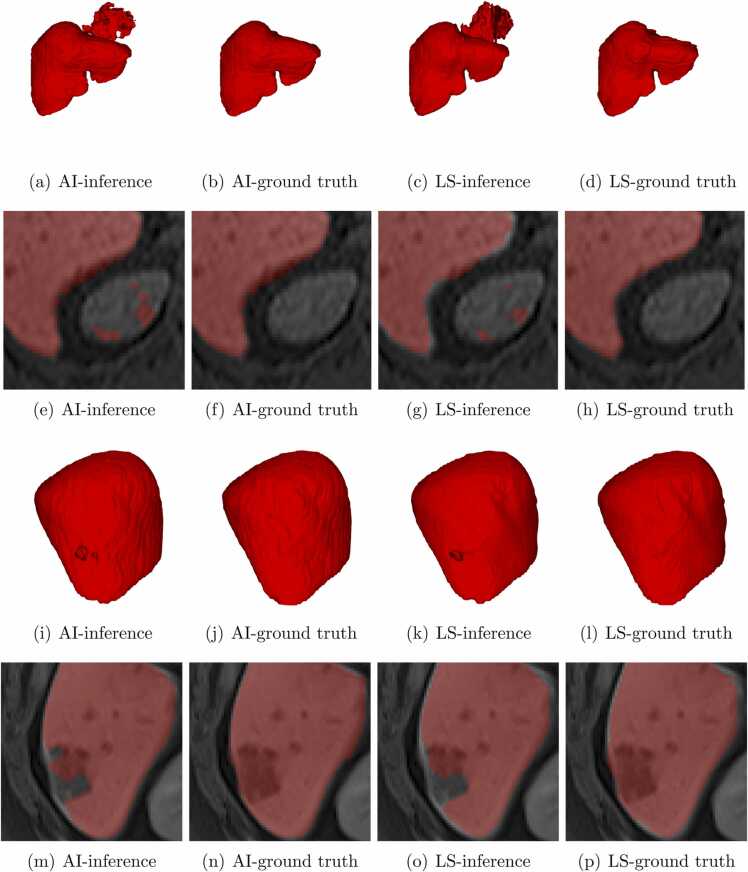
Examples of deep learning ((a), (e), (i), (m)), level set post-processed results ((c), (g), (k), (o)), and their corresponding manual corrections (((b), (f), (j), (n)) and ((d), (h), (l), (p))).

The clinical evaluation dataset, a total of 19 clinical cases of MR images, and its deep learning and level set post-processed deep learning segmentations were presented to the review team. During the meeting, participants requested to view 3D liver models colored red on a black background rather than a bright green, which made some of the surfaces less appealing. Examinations of 3D deep learning and level set post-processed segmentations show a large OS that concurs with surrounding organs. These types of segmentations are considered bad and distracting segmentations, and not suitable for clinical use without manual editing, shown in Figs. 5(a) and 5(c). In the overlay of segmentations on MR images, the axial plane confirmed various segmentation leaks into nearby organs such as the heart, spleen, stomach, and right kidney, shown in Figs. 5(e) and 5(g). The larger indents and holes on the liver surface were associated with under-segmentation (Figs. 5(m) and 5(o)), and most likely a missed lesion, although needed confirmation by viewing segmentation overlaid on an axial plane.

The participants from the clinical analysis meeting commented that some of the deep learning segmentations look more natural with a bit of a rough surface compared to the overly smooth surface of level set processed 3D models. In other cases, some larger granularity on the liver surface was removed through level set post-processing and improved liver surface. On the axial plane, determining edges of the liver parenchyma varied in quality between cases, and between deep learning inferences and level set post-processed segmentation although no significant and systematic difference has been observed.

Clinically most significant segmentation flaws were fully or partly missed lesions which were automatically categorized as incomplete segmentation and required corrections to include the whole lesion. In certain cases, the vast majority of the liver segmentations were perfect although part of the lesion has been missed. Therefore it requires some local correction for clinical acceptance or standard segmentations. In seven cases (37%), deep learning and level set processed deep learning segmentations possess all required anatomical structures as well as pathology. These liver segmentations have some OS and US though a clear understandable liver shape included necessary vessels and lesions. Therefore after manual, semi-automatic, or even automatic separation of lesions and liver vessels inside of these segmentations, these inferences could be used for surgery planning without any modifications.

#### 3.3.2 Segmentation correction time

The deep learning inferences of the clinical evaluation dataset of 19 volumes were considered as initial segmentations and corrected manually to create clinical standard segmentations (AI-GT). The median time to complete a segmentation was 18 min (inter-quartile range: 13:18–22:46). After five weeks, the same cases of level set post-processed deep learning segmentations (LS) were corrected manually (LS-GT) with a median time was 16 min (inter-quartile range: 12:47–21:56).

To evaluate the segmentation quality of the proposed method, we evaluated the results of both deep learning and level set results against AI-GT and LS-GT. Table 3 shows the impact of these corrections in terms of evaluation metrics values. The values for (AI-GT)+ (LS-GT) are obtained using “logical or” operation, and (AI-GT). (LS-GT) is obtained using Hadamard products of AI-GT and LS-GT. Intra-observer variation was measured to be about 3% in the Dice score between AI-GT and LS-GT. The HD_95_ is 1.6 mm for the AI-GT versus LS-GT which is ten times lesser than automatic method predictions and their corresponding manual corrections.

**Table 3:** Comparison of clinical evaluation dataset of 19 volumes with manually corrected AI and LS results.

Evaluations	Dice	JAC	OS	US	HD_95_
AI vs AI-GT	0.9540 ± 0.0561	0.917 ± 0.0958	0.0622 ± 0.1015	0.0297 ± 0.0413	14.5142 ± 20.6775
AI vs (AI-GT).(LS-GT)	0.9392 ± 0.0586	0.8905 ± 0.0972	0.0975 ± 0.1092	0.0241 ± 0.0385	14.7683 ± 21.0661
AI vs (AI-GT)+ (LS-GT)	0.9497 ± 0.0540	0.9087 ± 0.0919	0.0589 ± 0.0982	0.0417 ± 0.0431	14.5266 ± 20.3651
LS vs LS-GT	0.9490 ± 0.0578	0.9081 ± 0.0981	0.0644 ± 0.1031	0.0376 ± 0.0443	15.0082 ± 20.6430
LS vs (AI-GT).(LS-GT)	0.9483 ± 0.0577	0.9068 ± 0.0977	0.0731 ± 0.1046	0.0303 ± 0.0412	15.1672 ± 21.0178
LS vs (AI-GT)+ (LS-GT)	0.9349 ± 0.0549	0.8822 ± 0.0917	0.0584 ± 0.0955	0.0718 ± 0.0468	15.0700 ± 20.2732
AI-GT vs LS-GT	0.9718 ± 0.0069	0.9453 ± 0.0130	N/A	N/A	1.5573 ± 0.3267

## 4 Discussion

Image-guided surgery systems have proven efficiency for navigation and excision in laparoscopic liver resection surgery planning along with the help of the latest techniques in segmentation, 3D model creation, registration, and tracking [11], [12]. To create segmentations we presented an approach for the automated segmentation of liver parenchyma in T1-weighted MR images using a deep learning algorithm with and without level set post-processing. Both the deep learning and level set results were used for the clinical validation study to analyze the impact of these automated methods.

We used UNet variants with/without attention gate and obtained optimal outcomes for the T1 sequences of MR images. The literature shows that the neural network model with attention gate learns the target structures of varying shapes and sizes, and better training convergence, etc. [47], [48]. The UNet_32_ model without attention gate shows highest Dice score of 0.9696 for Tversky loss with *α* = 0.1 and *β* = 0.9. On the other hand, Salehi et al. [49] showed Tversky loss with *α* = 0.3 and *β* = 0.7 shows better result in their study. It is one of the important predictions from our experiment that there is a possibility to obtain a better performance depending on the choice of parameter choices in Tversky loss, the dataset, the network, etc.

In the level set experiment, we initiated our experiments with the base values from Li et al. [42] and experimented on the inner and outer iteration counts for level set evolution with different *α* and *λ* values. If the boundary appears to be with weak edges, a higher *α* value may cause leakage. The liver and its other neighboring organs such as the heart, blood vessels, etc., can have hyper-intense values due to contrast injection that may cause weak edges. Though it is difficult to determine generalized values of the control parameters that yields optimal segmentation result, we made an effort to determine them by conducting experiments with many possible parameter choices and evaluating metric scores.

In Fig. 2, volume 4 in the test dataset represents both the AI and LS segmentation results that appear to be as close as the ground truth. Also, the image slices corresponding to the third row in Fig. 2 the show effectiveness of the level set in the case of the US. In comparison (yellow colored circles) with the ground truth image (Fig. 2(c)), the deep learning algorithm failed to segment some pixels in the boundary (Fig. 2(g)), and the level set algorithm correctly segment those missed pixels by AI (Fig. 2(k)). We have also observed that the level set fails if there is less connectivity in pixels. For example, the ground truth (Fig. 2(d)) shows a weak connectivity region, the deep learning result (Fig. 2(h)) loses its connection, and the level set result (Fig. 2(l)) appears to be completely lost (yellow colored circles).

In Fig. 3, volume 24 in the test dataset represents deep learning, and the level set result shows OS with leakage into the boundary and connecting region (yellow-colored circles). The metric scores of volume 24 in the test dataset (Table 2) show that the level set achieved lower OS, and Dice scores compared to deep learning results. Contrary to our expectations, the use of the level set method did not enhance the Dice score performance of deep learning results. The level set parameters were predetermined in this work to have an automatic process, but a semi-automatic level set might have given better results.

In general, leakage in boundary pixels is predominant and this drives less attention toward examining the inner pixel accuracy. We applied 5 × 5 × 5 and 3 × 3 × 3 voxels border removal to assess the effectiveness of the used AI and level set algorithms in terms of OS and US. Thus border removal helps to asses directly the most important inner regions. Fig. 4 shows the comparison of metrics distribution for AI, LS, AI5, and LS5. This approach enhanced the metrics of OS and US. The substantial impact could be seen in Dice and US scores, whereas the OS and HD remain less affected.

**Fig. 6:**
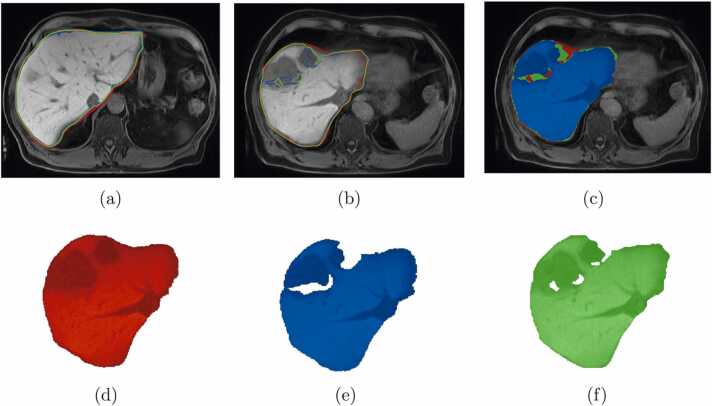
Examples of (a) contours of best Dice score result overlaid, (b) contours of worst Dice score result overlaid, (c) ground truth, deep learning, and level set segmentation overlaid, (d) ground truth (red) and their corresponding worst 2D slice results for (e) deep learning (blue), (f) level set results (green).

A visual inspection of AI or LS inferences overlaid with the ground truth can explain the over and under segmentations, for which we considered volume number 2 of the test dataset where both AI and level set algorithms failed to predict tumor region. The visual inspection shows that the contours of AI and LS inferences are consistent with ground truth in some slices as shown in Fig. 6(a). However, the network fails to predict the whole large lesion region, and major corrections are still needed after level set post-processing, shown in Fig. 6(b). The analysis of results from the same volume on multiple slices shows that our method fails to classify tumor regions that appear at the periphery of the liver, as shown in Fig. 6(c).

The visual analysis by clinicians The analysis of segmented 3D models from clinical evaluation shows that both the deep learning and level set segmentation required manual correction in 12 cases. The clinical acceptance of both deep learning and level set results varied between cases but overall the clinicians suggest the practical usage of inference results, after additional anatomical structure separation, in surgical planning.

After five weeks (to avoid bias) of the manual corrections of deep learning predictions, the same clinician performed manual corrections on level set predictions. The variability was measured in terms of deep learning and level set results versus their corrections. We have observed an intra-observer variability of 3% between deep learning and level set results corrected segmentations. We also evaluated metrics on these corrected segmentations (see Table 3). The correction time required for level set predictions is 2 min median time less than the correction time of deep learning predictions. It is difficult to make direct comparisons with other works in literature since different approaches, datasets, and delineation criteria were used in various published studies.

The time analysis to create clinically acceptable segmentation by manual correction of AI and level set inferences were carried out using 19 volumes of MR images by an appropriately trained medical doctor. A comparison with fully manually segmented MR images would be of value though would be challenging on a such scale. Firstly, a group of trained radiologists or medical doctors need to create manual segmentation for the test set. Secondly, AI inferences of the same images need to be corrected manually. Finally, level set inferences of the same images need to be corrected manually. It is a three-fold work with the same test dataset, and it would include many hours for complete manual segmentation and correction. The current work focuses on AI segmentation of liver parenchyma and not the full organ, and it would be even more time-consuming. Therefore a smaller scale may be planned in the future with multiple labels (parenchyma, separated vessels, and potentially liver lesions). The complete workflow evaluation could also be tested in the future measuring time needed from image acquisition to full liver segmentation approved by a clinician. This would be very relevant to working towards the use of intra-operative CT for liver resection planning and volumetric analysis e.g. living donor.

In this work, we used 256 resampled volumes as the input to the network, and scores were measured with the original data shapes. Our proposed AI algorithm performs better in terms of evaluation scores compared to other methods. Using different networks with RAM and Video RAM memory constraints, loss functions, highly variable data, network design, contrast enhancement pre-processing, etc., could lead to better results. The use of a 2D-slice-based level set for post-processing results in a stacked visualization of slice segmentation in the 3D models, which could be managed by using the volume-based level set approach. Apart from this, the use of GPU computation instead of CPU-based level set segmentation could reduce the time taken. Also, a potential way to avoid intra-observer variations to a certain level is by involving multiple clinical experts to correct the segmentations, which is however difficult as more time needs to be spent by clinicians on segmentations.

## 5 Conclusion

In this paper, we have analyzed the clinical applicability of two automated (deep learning and level set) algorithms for liver parenchyma segmentation of contrast-enhanced T1-weighted magnetic resonance (MR) images. The dataset possesses a certain degree of variability, which includes two different MR machine manufacturers with seven models. We have achieved the highest dice score of 0.9696 when compared to state-of-the-art deep learning algorithms in liver parenchyma segmentation. We have proved that the Tversky loss achieves higher scores other than the standard parameters. Apart from this, the importance of this research work also includes an analysis of clinical acceptance of the created segmentations, 3D models, and the time to create clinically accepted segmentations. We addressed some of the segmentation difficulties including missing tumor regions, boundary leakage, and over-segmentation of AI-based liver parenchyma segmentation by combining the merits of the deep learning algorithm with a level set approach. Our experiments show that approximately one-third of the segmentations using automated algorithms can produce results without the need for manual correction. Further research directions in this work may include a complete segmentation with lesions, vascular structures, and clinical applicability with a use case for surgery resection study.

## CRediT authorship contribution statement

**Varatharajan Nainamalai:** Conceptualization, Methodology, Software, Validation, Formal analysis, Resources, Investigation, Data curation, Visualization, Supervision, Writing - original draft, Writing - review & editing. **Pravda Jith Ray Prasad:** Conceptualization, Methodology, Software, Validation, Formal analysis, Investigation, Data curation, Visualization, Writing - original draft, Writing - review & editing. **Egidijus Pelanis:** Conceptualization, Methodology, Software, Validation, Formal analysis, Resources, Investigation, Data curation, Writing – original draft, Visualization, Writing – review & editing. **Bjørn Edwin:** Data curation, Supervision, Project administration, Funding acquisition, Investigation, Methodology, Resources, Validation, Writing - review & editing. **Fritz Albregtsen:** Formal analysis, Conceptualization, Supervision, Writing - review & editing. **Ole Jakob Elle:** Supervision, Project administration, Funding acquisition, Resources, Writing - review & editing. **Rahul P. Kumar:** Conceptualization, Supervision, Project administration, Writing - review & editing.

## Declaration of Competing Interest

The authors declare that they have no known competing financial interests or personal relationships that could have appeared to influence the work reported in this paper.
